# Identification of HLA-DRB1*04:*10* allele as risk allele for Japanese moyamoya disease and its association with autoimmune thyroid disease: A case-control study

**DOI:** 10.1371/journal.pone.0220858

**Published:** 2019-08-14

**Authors:** Ryosuke Tashiro, Kuniyasu Niizuma, Seik-Soon Khor, Katsushi Tokunaga, Miki Fujimura, Hiroyuki Sakata, Hidenori Endo, Hidetoshi Inoko, Koetsu Ogasawara, Teiji Tominaga

**Affiliations:** 1 Department of Neurosurgery, Tohoku University Graduate School of Medicine, Sendai, Japan; 2 Department of Immunobiology, Tohoku University Institute of Development, Aging and Cancer, Sendai, Japan; 3 Department of Neurosurgical Engineering and Translational Neuroscience, Graduate School of Biomedical Engineering, Tohoku University, Sendai, Japan; 4 Department of Neurosurgical Engineering and Translational Neuroscience, Tohoku University Graduate School of Medicine, Sendai, Japan; 5 Department of Human Genetics, the University of Tokyo Graduate School of Medicine, Tokyo, Japan; 6 Department of Neurosurgery, Kohnan Hospital, Sendai, Japan; 7 Genodive Pharma Inc., Atsugi, Kanagawa, Japan; Centro Cardiologico Monzino, ITALY

## Abstract

**Background and purpose:**

Moyamoya disease (MMD) is a progressive cerebrovascular disease with unknown etiology. Growing evidence suggest its involvement of autoimmune and genetic mechanisms in the pathogenesis of MMD. This study aims to clarify the association between *HLA* allele and MMD.

**Methods:**

Case-control study: the DNA of 136 MMD patients in Japan was extracted and the genotype of *human leukocyte antigen* (*HLA*) from this DNA was determined by super-high-resolution single-molecule sequence-based typing using next-generation sequencing. Next, the frequency of each *HLA* allele (*HLA-A*, *HLA-B*, *HLA-C*, *HLA-DRB1*, *HLA-DQB1*, and *HLA-DPB1*) was compared with those in the Japanese control database. In addition, haplotype estimation was performed using the expectation maximization algorithm.

**Results:**

The frequencies of the HLA-DRB1*04:*10* allele (4.77% vs. 1.47% in the control group; *P* = 1.7 × 10^−3^; odds ratio [OR] = 3.35) and of the HLA-DRB1*04:10–HLA-DQB1*04:*02* haplotype (haplotype frequency 4.41% vs. 1.35% in the control group; *P* = 2.0 × 10^−3^; OR = 3.37) significantly increased. The frequency of thyroid diseases, such as Graves’ disease and Hashimoto thyroiditis, increased in HLA-DRB1*04:*10*-positive MMD patients compared with that in HLA-DRB1*04:*10*-negative MMD patients.

**Conclusions:**

HLA-DRB1*04:*10* is a risk allele and HLA-DRB1*04:10–HLA-DQB1*04:*02* a risk haplotype for MMD. In addition, HLA-DRB1*04:*10* is associated with thyroid disease in MMD patients.

## Introduction

Moyamoya disease (MMD) is a chronic, occlusive cerebrovascular disease with unknown etiology. MMD involves bilateral steno-occlusive changes at the terminal portion of the internal carotid artery and the formation of an abnormal vascular network at the base of the brain [1]. MMD is a relatively rare disease, as estimated incidence of MMD is 3.1–10.5/100,000 in Japanese population [2–3]. Despite its rarity, MMD is one of the leading causes of cerebrovascular diseases among children and young adults in Japan and Korea. Thus, it is expected to unveil the pathogenesis of MMD and diagnose MMD before onset of stroke. Although the etiology of MMD remains undetermined, previous studies showed an association between autoimmunity and MMD [4–11], such as the high frequencies of thyroid dysfunction and type 1 diabetes among MMD patients [4–10].

Human leukocyte antigen (HLA) proteins provide peptides to T-cell receptors for the maintenance of self-tolerance and adapted immunity [12–14]. Allelic and haplotypic differences between *HLA* alleles are associated mainly with human autoimmune diseases [13–15]. However, despite accumulating evidence, it is unclear how genetic variations of *HLA* alleles lead to a risk of autoimmune diseases. Recent studies have analyzed *HLA*–peptide–T-cell receptor complexes and demonstrated the autoantigen recognition mechanism [15–17]. Therefore, to understand the autoimmunity mechanism, it is critical to determine *HLA* alleles susceptible and/or resistant to autoimmune diseases.

Several studies have demonstrated the association between MMD and allelic and haplotypic differences of *HLA*, including both *HLA* class I and class II alleles (e.g., HLA-A*24, HLA-B*54, HLA-DRB1*04:*05*, and HLA-DQB1*04:*01*) in multiple cohorts of East Asians [18–21], as well as in Europeans [22]. We sought to determine susceptible allele for MMD using next-generation sequence (NGS), since conventional *HLA* DNA typing methods, including sequence-based typing (SBT) and sequence-specific oligonucleotide (SSO) typing, yield ambiguous results because of oligonucleotide probe design limitations or phase ambiguity for *HLA* allele assignment [23]. The recently developed super-high-resolution single-molecule sequence-based typing (SS-SBT) method using NGS can determine an *HLA* allele sequence at the 8-digit level and also overcome the phase ambiguity of earlier typing methods [24].

In this study, we analyzed the association between MMD and *HLA* alleles using SS-SBT and NGS in Japanese MMD patients.

## Materials and methods

### Study design

This is a case-control study. MMD patient samples were collected at Tohoku University hospital and Kohnan hospital. We referred *HLA* database of the University of Tokyo as for healthy control. Based on previous reports demonstrating *HLA* alleles and autoimmune diseases [13–15], we calculated the number of samples to be enrolled in this study as more than 130 MMD patients and 300–500 healthy controls, respectively. Written informed consent for participation in the study was obtained from all subjects and (in the case of pediatric patients) from their guardians. After obtaining written informed consent, we collected blood samples and performed *HLA* genotyping. Hardy-Weinberg equation (HWE) was not considered in this study, since previous reports demonstrated deviation from HWE for major histocompatibility region on chromosome 6 [25]. The study complied with the Helsinki Declaration on ethical principles for medical research involving human subjects. Ethical approval was obtained from the ethical committees of Tohoku University, Kohnan Hospital, and the University of Tokyo.

### Subject population

All MMD patients enrolled in the study (*n* = 136, aged 11–78 years, mean 43.1) met the diagnostic criteria established by the Research Committee on Spontaneous Occlusion of the Circle of Willis, the Ministry of Health, Labor and Welfare, Japan [26]. Patients with quasi- moyamoya disease were not enrolled in this study. Samples were collected between 2016 and 2018, and most of the patients were residents in Tohoku region in Japan. Clinical characteristics of enrolled patients were extracted from the databases in Tohoku University Hospital and Kohnan hospital.

### *HLA* genotyping

We performed *HLA* genotyping with SS-SBT at the 8-digit level using NGS, as reported in previous studies [24]. Outline of procedures is shown in https://dx.doi.org/10.17504/protocols.io.3rxgm7n [PROTOCOL DOI]. Briefly, genomic DNA was obtained from the patients’ 2ml of whole blood using the QIAamp DNA Mini Kit for genomic DNA purification (Qiagen GmbH, Hilden, Germany), and 400 ng of purified genomic DNA was used for polymerase chain reaction (PCR) amplification. DNA was preserved in 4°C freezer. The basic cycling parameters were as follows: (i) first denaturation at 94°C for 2 min, followed by 30 cycles of denaturation at 98°C for 10 s and 60°C for 20 s and extension at 68°C for 5 min (*HLA-A*, *HLA-B*, and *HLA-C*); (ii) first denaturation at 94°C for 2 min, followed by 30 cycles of denaturation at 98°C for 10 s and annealing at 70°C for 5 min (*HLA-DRB1* and *HLA-DPB1*); and (iii) first denaturation at 94°C for 2 min, followed by 30 cycles of denaturation at 98°C for 10 s and annealing at 70°C for 9 min (*HLA-DQB1*). Long-range PCR reactions were performed using the thermal cycler Gene Amp PCR System 9700 (Life Technologies, Carlsbad, CA, USA). The PCR products obtained were purified with Agencourt AMPure XP (Beckman Coutler, CA, USA) and quantified by the Quant-iT Picogreen dsDNA Assay Kit (Thermo Fisher Scientific, MA, USA). Next, the PCR products were clonally amplified and barcoded using the Ion Plus Fragment Library Kit (Life Technologies), and the barcoded library was sequenced using the Ion Torrent Personal Genome Machine DNA sequencing system (Life Technologies). The NGS read data were analyzed by Sequence Alignment Based Assigning Software (SeaBass), and finally, the *HLA* alleles were determined.

### Control database

We referred HLA database of the University of Tokyo. Control group does not include patients with MMD and autoimmune disease. Control samples have been collected between 1990 and 2018, and most of the healthy controls were residents in Kanto district in Japan. *HLA* genotyping was performed, as reported previously [27–28]. *HLA* class I (*HLA-A*, *HLA-B*, and *HLA-C*) and class II (*HLA-DRB1*, *HLA-DQB1*, and *HLA-DPB1*) genotypes were determined with the HLA-DNA Typing Kit, Luminex multianalyte profiling system xMAP (Liminex Corp., Austin, TX, USA), WAKFlow HLA Typing Kit (Wakunaga Pharmaceutical, Osaka, Japan), and LABType SSO HLA Kit (One Lambda, Canoga Park, CA, USA) according to the manufacturers’ instructions. All *HLA* alleles were determined at the 4-digit level.

### Haplotype estimation

We performed 2-locus (*HLA-DRB1–HLA-DQB1* and *HLA-A–HLA-B*) and 3-locus (*HLA-DR1–HLA-DQB1–HLA-DPB1* and *HLA-A–HLA-B–HLA-C*) haplotype analysis using Bridging ImmunoGenomic Data-Analysis Workflow Gaps (BigDAWG) [29]. The pairwise linkage disequilibrium (LD) parameters, *r*^2^ and *D*’, between alleles at different class II *HLA* loci were calculated on the basis of the haplotype frequencies estimated by the expectation maximization (EM) algorithm [30–31].

### Determination of polymorphisms in the *RNF213* gene

Polymorphisms in the *RNF213* gene was determined as previously reported [32]. Single nucleotide polymorphism (SNP) for rs112735431 (https://www.ncbi.nlm.nih.gov/projects/SNP/snp_ref.cgi?rs=112735431) was analyzed using the TaqMan SNP genotyping assay (Assay ID: C_153120198_10; Applied Biosystems, Foster City, CA, USA) on a StepOnePlus real-time polymerase chain reaction (PCR) system (Applied Biosystems, Foster City, CA, USA). The basic cycling parameters were as follows: hold at 95°C for 10 minutes, followed by 40 cycles of PCR amplification comprising of denaturation at 95°C for 15 seconds, and annealing and extension at 60°C for 1 minute. Genotype calls were evaluated with Applied Biosystems TaqMan Genotype software. The investigators who assessed genotype were blinded to phenotypic information.

### Statistical analysis

Statistical tests were performed using JMP Pro version 13 (SAS Institute). For each testing scenario, prior to statistical analysis, rare alleles (expected counts < 5) were combined into the “Others” category. The differences between allele and haplotype frequencies were calculated by the chi-square test. Adjustment for multiple comparisons was conducted using the Bonferroni method. Corrected *P*-values (*P*_c_) were calculated by multiplying the *P*-values with the number of examined alleles. The association between HLA-DRB1*04:*10* and clinical characteristics was evaluated using Mann–Whitney’s U test, and assessment of continuous variables and categorical variables was performed using Fisher’s exact test the chi-square test, respectively. Quantitative variables, such as Suzuki stage and age at onset, were stratified into several categories. For example, age at onset was stratified into three categorical variables, eg, child-onset, elderly-onset (age > 60), perinatal period-onset. Multivariate logistic analysis using Poisson regression models was used to adjust for age and sex. *P*-values of <0.05 were considered statistically significant.

### Compliance with STrengthening the REporting of Genetic Association Studies (STREGA) Guidelines

This study complied with STrengthening the REporting of Genetic Association Studies (STREGA) Guidelines [33], as shown in **S1 Table**.

## Results

### HLA-DRB1*04:*10* is a risk allele for MMD

In this study, we examined the frequencies of *HLA* class I (*HLA-A*, *HLA-B*, and *HLA*-*C*) and class II (*HLA-DRB1*, *HLA-DQB1*, and *HLA-DPB1*) alleles in MMD patients and controls. **Tables** 1–3 shows the frequencies for *HLA* class II alleles in MMD patients and controls. As seen in the table, HLA-DRB1*04:*10* was significantly associated with MMD: allele frequency 4.77% vs. 1.47% in the control group; *P* = 1.7 × 10^−3^; odds ratio [OR] = 3.35. The frequency of HLA-DRB1*04:*06* decreased in MMD patients compared with that in the control group: allele frequency 1.10% vs. 3.44% in the control group; *P* = 0.045; OR = 0.31. However, after Bonferroni correction, the *P*-value (*P*_c_) did not reach the threshold (**Table 1**). In addition, we found no association between other *HLA* class II alleles (*HLA-DQB1* and *HLA-DPB1*) and MMD (**Tables 2 and 3**) and no association between *HLA* class I alleles (*HLA-A*, *HLA-B*, and *HLA*-*C*) and MMD (**S2 Table**).

**Table 1:** Frequencies of *HLA-DRB1* allele carrier in MMD patients and controls.

Locus	Allele	Control (2n = 814)	Patient (2n = 272)	OR (95%CI)	P value	P_c_
DRB1	1:01	7.00 (57)	5.88 (16)	0.83 (0.44–1.50)	0.523	NS
DRB1	4:03	2.95 (24)	2.57 (7)	0.87 (0.31–2.11)	0.748	NS
DRB1	4:05	14.4 (117)	10.3 (28)	0.68 (0.42–1.07)	0.087	NS
DRB1	4:06	3.44 (28)	1.10 (3)	0.31 (0.06–1.03)	0.045	NS
DRB1	4:10	1.47 (12)	4.77 (13)	3.35 (1.39–8.14)	1.7 x 10^−3^	0.030
DRB1	8:02	3.81 (31)	5.15 (14)	1.37 (0.66–2.70)	0.338	NS
DRB1	8:03	7.62 (62)	8.09 (22)	1.07 (0.61–1.80)	0.801	NS
DRB1	9:01	14.9 (121)	18.0 (49)	1.26 (0.85–1.83)	0.216	NS
DRB1	11:01	2.83 (23)	1.84 (5)	0.64 (0.19–0.76)	0.374	NS
DRB1	12:01	3.56 (29)	3.31 (9)	0.93 (0.38–2.04)	0.844	NS
DRB1	12:02	2.21 (18)	1.47 (4)	0.66 (0.16–2.03)	0.453	NS
DRB1	13:02	7.62 (62)	6.25 (17)	0.81 (0.43–1.43)	0.452	NS
DRB1	14:05	1.97 (16)	1.84 (5)	0.93 (0.27–2.70)	0.895	NS
DRB1	14:06	1.60 (13)	2.94 (8)	1.87 (0.66–4.92)	0.163	NS
DRB1	14:54	3.19 (26)	1.47 (4)	0.45 (0.11–1.32)	0.133	NS
DRB1	15:01	8.11 (66)	6.25 (17)	0.76 (0.41–1.33)	0.318	NS
DRB1	15:02	8.60 (70)	10.3 (28)	1.22 (0.74–1.97)	0.398	NS
DRB1	others	4.79 (39)	8.46 (23)	1.84 (1.02–3.22)	0.024	NS

The number of allele carriers is shown in parentheses. The association was examined by the chi-square test. Corrected *P*-values (*P*_c_) statistically significant after Bonferroni correction are indicated in bold. OR, odds ratio; CI, confidence interval; *P*_c_, corrected *P-*value; NS, not significant.

**Table 2:** Frequencies of *HLA-DQB1* allele carrier in MMD patients and controls.

locus	Allele	Control (2n = 814)	Patient (2n = 272)	OR (95%CI)	P value	P_c_
DQB1	3:01	12.0 (98)	14.3 (39)	1.22 (0.80–1.85)	0.323	NS
DQB1	3:02	9.34 (76)	9.19 (25)	0.98 (0.59–1.60)	0.943	NS
DQB1	3:03	15.6 (127)	18.4 (50)	1.22 (0.83–1.77)	0.282	NS
DQB1	4:01	14.4 (117)	9.56 (26)	0.63 (0.39–1.00)	0.042	NS
DQB1	4:02	3.19 (26)	6.99 (19)	2.28 (1.17–4.35)	0.006	NS
DQB1	5:01	7.74 (63)	6.62 (18)	0.84 (0.46–1.48)	0.542	NS
DQB1	5:02	2.09 (17)	1.47 (4)	0.70 (0.17–2.17)	0.522	NS
DQB1	5:03	3.56 (29)	2.94 (8)	0.82 (0.32–1.87)	0.625	NS
DQB1	6:01	16.2 (132)	18.0 (49)	1.14 (0.77–1.65)	0.491	NS
DQB1	6:02	7.86 (64)	5.88 (16)	0.73 (0.39–1.31)	0.279	NS
DQB1	6:04	7.37 (60)	6.25 (17)	0.84 (0.45–1.49)	0.533	NS
DQB1	others	0.62 (5)	0.37 (1)	0.60 (0.01–5.37)	0.635	NS

The number of allele carriers is shown in parentheses. The association was examined by the chi-square test. Corrected *P*-values (*P*_c_) statistically significant after Bonferroni correction are indicated in bold. OR, odds ratio; CI, confidence interval; *P*_c_, corrected *P-*value; NS, not significant.

**Table 3:** Frequencies of *HLA-DPB1* allele carrier in MMD patients and controls.

locus	Allele	Control (2n = 814)	Patient (2n = 272)	OR (95%CI)	P value	P_c_
DPB1	2:01	25.2 (205)	29.4 (80)	1.24 (0.90–1.69)	0.170	NS
DPB1	2:02	4.30 (35)	1.84 (5)	0.42 (0.13–1.08)	0.062	NS
DPB1	3:01	4.42 (36)	4.41 (12)	1.00 (0.42–2.00)	0.994	NS
DPB1	4:01	6.02 (49)	4.41 (12)	0.72 (0.34–1.40)	0.319	NS
DPB1	4:02	10.1 (82)	8.09 (22)	0.79 (0.46–1.30)	0.335	NS
DPB1	5:01	38.0 (309)	36.4 (99)	0.94 (0.70–1.25)	0.645	NS
DPB1	9:01	7.86 (64)	9.93 (27)	1.29 (0.77–2.11)	0.287	NS
DPB1	others	4.18 (34)	5.52 (15)	1.34 (0.67–2.57)	0.357	NS

The number of allele carriers is shown in parentheses. The association was examined by the chi-square test. Corrected *P*-values (*P*_c_) statistically significant after Bonferroni correction are indicated in bold. OR, odds ratio; CI, confidence interval; *P*_c_, corrected *P-*value; NS, not significant.

### Association between the *HLA-DR1–HLA-DQB1* haplotype and MMD

**Table 4** and **S2 Table** show the haplotype frequency estimated using the EM algorithm. The frequency of HLA-DRB1*04:10–HLA-DQB1*04:*02* increased in MMD patients (**Table 4**): haplotype frequency 4.41% vs. 1.35% in the control group; *P* = 2.0 × 10^−3^; OR = 3.37. On the other hand, the frequencies of HLA-DRB1*04:05–HLA-DQB1*04:*01* (haplotype frequency 9.56% vs. 14.3% in the control group; *P* = 0.047; OR = 0.64) and HLA-DRB1*04:06–HLA-DQB1*03:*02* (haplotype frequency 1.10% vs. 3.44% in the control group; *P* = 0.045; OR = 0.31) decreased in MMD patients. However, after Bonferroni correction, the *P*-value (*P*_c_) did not reach the threshold (**Table 4**). In addition, we found no association between *HLA-DRB1–HLA-DQB1–HLA-DPB1*, *HLA-A–HLA-B*, and *HLA-A–HLA-B–HLA-C* haplotypes and MMD (**S3 Table**).

**Table 4:** Frequencies of estimated *HLA-DRB1-DQB1* haplotype carrier in MMD patients and controls.

DRB1/DQB1	Control (2n = 814)	Patient (2n = 272)	OR (95%CI)	P value	P_c_
*01:01-*05:01	7.00 (57)	5.88 (16)	0.83 (0.44–1.50)	0.523	NS
*04:03-*03:02	2.83 (23)	2.57 (7)	0.91 (0.33–2.22)	0.826	NS
*04:05-*04:01	14.3 (116)	9.56 (26)	0.64 (0.39–1.01)	0.047	NS
*04:06-*03:02	3.44 (28)	1.10 (3)	0.31 (0.06–1.03)	0.045	NS
*04:10-*04:02	1.35 (11)	4.41 (12)	3.37 (1.34–8.53)	0.002	0.036
*08:02-*03:02	1.97 (16)	2.94 (8)	1.51 (0.55–3.79)	0.343	NS
*08:02-*04:02	1.84 (15)	2.21 (6)	1.20 (0.38–3.32)	0.707	NS
*08:03-*06:01	7.62 (62)	7.72 (21)	1.01 (0.58–1.73)	0.955	NS
*09:01-*03:03	14.4 (117)	17.3 (47)	1.24 (0.84–1.82)	0.247	NS
*11:01-*03:01	2.58 (21)	1.84 (5)	0.71 (0.21–1.95)	0.488	NS
*12:01-*03:01	2.58 (21)	2.94 (8)	1.14 (0.43–2.73)	0.749	NS
*12:02-*03:01	2.21 (18)	1.47 (4)	0.66 (0.16–2.03)	0.453	NS
*13:02-*06:04	7.37 (60)	6.25 (17)	0.84 (0.45–1.49)	0.533	NS
*14:05-*05:03	1.84 (15)	1.84 (5)	1.00 (0.28–2.92)	0.996	NS
*14:06-*03:01	1.60 (13)	2.94 (8)	1.87 (0.66–4.92)	0.163	NS
*15:01-*06:02	7.86 (64)	5.88 (16)	0.73 (0.39–1.31)	0.279	NS
*15:02-*06:01	8.60 (70)	10.3 (28)	1.22 (0.74–1.97)	0.398	NS
Others	10.7 (87)	12.9 (35)	1.23 (0.79–1.90)	0.324	NS

The number of haplotype carriers estimated by the EM algorithm is shown in parentheses. The association was examined by the chi-square test. Corrected *P*-values (*P*_c_) statistically significant after Bonferroni correction are indicated in bold.

*, Separator; OR, odds ratio; CI, confidence interval; *P*_c_, corrected *P-*value; NS, not significant.

### High frequency of thyroid dysfunction in HLA-DRB1*04:*10*-positive MMD patients

**Table 5** shows the results of our analysis of the association between HLA-DRB1*04:*10* and MMD clinical characteristics. The results indicated that the frequency of thyroid diseases, including Graves’ disease (GD) and Hashimoto thyroiditis (HT), is high in HLA-DRB1*04:*10*-positive MMD patients compared with that in HLA-DRB1*04:*10*-negative MMD patients (frequency 23.1% vs. 4.1% in the control group; *P* = 0.029). In addition, the HLA-DRB1*04:*10*-positive group had more females (frequency 92.3% vs. 75.6%; *P* = 0.297), younger patients (mean age of onset 30.1 ± 13.3 vs. 35.4 ± 16.0 years in the control group; *P* = 0.206), and higher incidences of hemorrhagic stroke (frequency 23.1% vs. 7.3% in the control group; *P* = 0.091) than the HLA-DRB1*04:*10*-negative group. However, these differences were not statistically significant. We also found no association among polymorphisms in the *ring finger protein 213* (*RNF213*) gene, Suzuki stage, and *HLA-DRB1* genotype. After adjustment for age and sex, significantly higher proportion of HLA-DRB1*04:*10*-positive MMD patients had thyroid disease compared with HLA-DRB1*04:*10*-negative MMD patients (*P* = 0.039).

**Table 5:** Comparison of demographics between MMD patients with or without HLA-DRB1*04:*10*.

			unadjusted		Adjusted	
	DRB1*04:*10* -positive (n = 13)	DRB1*04:*10* -negative (n = 123)	OR (95%CI)	P value	OR (95%CI)	P value
Age	38.8 ± 10.8	43.6 ± 15.4		0.258†		
Sex (Male: Female)	1:12	30:93	0.26 (0.03–2.07)	0.297†		
History						
Diabetes	0% (0)	0.8% (1)	0	0.744†^ab^		
Thyroid diseases	23.1% (3)	4.1% (5)	7.08 (1.47–34.0)	0.029†	5.61 (1.09–28.9)	0.039
Other autoimmune diseases	0% (0)	0% (0)	0	1.000†		
Polymorphism c.14576G>A in *RNF213* gene				0.784§		
G/G	38.5% (5)	30.1% (38)	1.40 (0.43–4.55)	0.549‡		
G/A	61.5% (8)	67.4% (83)	0.77 (0.24–2.51)	0.771‡		
A/A	0% (0)	1.6% (2)	0	1.000‡		
Suzuki stage				0.187§		
1–2	15.4% (2)	27.6% (34)		0.513‡		
3–4	86.4% (11)	60.2% (74)				
5–6	0% (0)	12.2% (15)				
The age of onset	30.1 ± 13.3	35.4 ± 16.0		0.206†		
Child	15.4% (2)	17.9% (22)	0.48 (0.11–1.75)	0.822‡		
Onset >60	0% (0)	4.1% (5)	3.64 (0.77–17.1)	0.459‡		
Perinatal period	0% (0)	4.9% (6)	0	0.415‡		
Symptom				0.134§		
Ischemia	76.9% (10)	88.7% (109)	0.43 (0.11–1.75)	0.428‡		
Hemorrhage	23.1% (3)	7.3% (9)	3.80 (0.88–16.3)	0.091‡		
Asymptomatic	0% (0)	4.1% (5)	0	0.459‡		

The association between MMD patients with or without *HLA-DRB1*

*04:*10* was examined using Mann–Whitney’s U test, Fisher’s exact test, or the chi-square test. The number of patients is shown in parentheses, and the percentages are based on the number of patients per category. Mean values are represented as mean ± standard deviation (SD). A multiple logistic regression model was used to adjust for age and sex.

† Mann–Whitney’s U test

‡ Fisher’s exact test

§ chi-square test. *RNF213*, *ring finger protein 213*.

## Discussion

In this study, we identified HLA-DRB1*04:*10* as a risk allele and HLA-DRB1*04:10–HLA-DQB1*04:*02* as a risk haplotype for MMD. This result was inconsistent with previous results, which demonstrates HLA-DRB1*13:*02*, DRB1*15:*01*, DQB1*06:*02* and DQB1*06:*09*, as risk allele for MMD in East Asians [19–21]. This inconsistency might be resulted from lack of rigorous statistical analysis and limited range of subject population to familial cases in previous studies. Considering Based on the fact that the frequency of HLA-DQB1*04:*02*, which is in LD with HLA-DRB1*04:*10* allele, is not increased compared with the control group, and HLA-DRB1*08:*02* in LD with HLA-DQB1*04:*02* is not increased in MMD patients [34], it is conceivable that HLA-DRB1*04:*10*, not HLA-DQB1*04:*02*, is a risk allele for MMD. In addition, some *HLA-DR-DQ* haplotypes, such as HLA-DR9-DQA1*03–HLA-DQB1*03:*03* and HLA-DR4-DQA1*03–HLA-DQB1*04:*01*, lead to the risk of type 1 diabetes and autoimmune endocrinopathies in the Japanese population [13–14]. Therefore, *HLA* class II alleles are considered to play a key role in autoimmune reactions and it is possible that autoimmune reactions related to HLA-DRB1*04:*10* might play a role in MMD pathogenesis, although future studies including large cohorts should be performed.

In this study, we demonstrated that the frequency of thyroid diseases, including GD and HT, is high in HLA-DRB1*04:*10*-positive MMD patients compared with that in HLA-DRB1*04:*10*-negative MMD patients, suggesting the relationship between the HLA-DRB1*04:*10* haplotype and MMD and autoimmune thyroid diseases. Many studies have reported the association between thyroid dysfunction and MMD [7–12], suggesting that elevated autoantibody concentrations and hyperthyroidism are associated with stenotic lesions in the terminal portion of the internal carotid artery and aggressive MMD presentations [7–12]. However, the underlying mechanism of this association remains undetermined. Although GD and HT are multifactorial autoimmune diseases [35–38], they show opposite phenotypes: GD is characterized by the production of thyroid-stimulating hormone receptor–stimulating antibodies, leading to hyperthyroidism, whereas HT is characterized by the apoptosis of thyrocytes, resulting in hypothyroidism. However, GD and HT share an immunological basis; in fact, some alleles, such as *HLA*-A*02:*07*, *HLA*-B1*35:*01*, *HLA*-B1*46:*01*, and *HLA*-DRB4*53:*01*, are shared between GD and HT [35–39]. Although the association of *HLA*-DRB1*04:*10* with thyroid dysfunction has never been reported, its association with Vogt–Koyanagi–Harada disease and idiopathic thrombocytopenic purpura is known [40–41]. Future studies are required to clarify the functional role of HLA-DRB1*04:*10* and the nature of its involvement in MMD pathogenesis, especially in patients with thyroid diseases.

The role of *RNF213* in autoimmune reactions is still unclear. Japanese researchers, including us, previously demonstrated that *RNF213* is a disease susceptibility gene for MMD [42–44]. The c.14576G>A polymorphism in *RNF213* was identified in 95% of familial MMD cases and 79% of sporadic MMD cases.^37,38^ The c.14576G>A polymorphism in *RNF213* is located in neither the AAA+ATPase domain nor the RING finger ubiquitin ligase domain [42–44]. Functional roles of *RNF213* and its polymorphism in MMD pathogenesis remain undetermined. However, since the c.14576G>A polymorphism in *RNF213* greatly enhances the risk of MMD pathogenesis and that patients with this polymorphism show earlier disease onset and more severe clinical manifestations than MMD patients without the polymorphism [44], it is conceivable that the c.14576G>A polymorphism in *RNF213* plays an important role in MMD pathogenesis. In a previous study, we reported regulatory T-cell decrease in *RNF213*-knockout mice [45]; therefore, *RNF213* might play a role in autoimmune reactions. In contrast, in this study, we found no association between HLA-DRB1*04:*10* and polymorphism in *RNF213* and no difference in the soluble CD163 and CXCL5 serum concentrations in MMD patients between the *RNF213* variant and normal variant groups [11]. These results suggested that the c.14576G>A polymorphism in *RNF213* might not directly affect antigen recognition and the function of antigen-presenting cells. Further research could clarify the involvement of the RNF213 protein in autoimmune reactions.

This study had a few limitations. HLA-DRB1*04:*10*-positive patients account for ~10% of Japanese MMD patients; therefore, its involvement should be investigated in a larger population or other institutions. In addition, association between thyroid dysfunction and MMD is different between Asians and Caucasians: GD is more prevalent among Japanese MMD patients, whereas HT is more prevalent among Caucasian MMD patients [5–10]. The patients and controls in this study were residents of Tohoku and Kanto districts. Although the distribution of *HLA* alleles in the Japanese population is relatively homogeneous, there might be a difference in the *HLA* allele frequencies in MMD patients living in other regions of Japan. Further studies, which includes more control and MMD patients from other institutions, are required to clarify the exact association of HLA-DRB1*04:*10* to MMD pathogenesis.

## Conclusions

This study revealed that HLA-DRB1*04:*10* and HLA-DRB1*04:10–HLA-DQB1*04:*02* are a risk allele and a risk haplotype, respectively, for MMD. In addition, the HLA-DRB1*04:*10* frequency increases in MMD patients with thyroid diseases. Although further studies are required to clarify the exact association between *HLA* class II and MMD, autoimmunity might explain MMD pathogenesis, at least in part.

## Supporting information

S1 Table Strengthening the Reporting of Genetic Association Studies (STREGA) checklist. (DOCX)

S2 Table Frequencies of *HLA* class I carrier in MMD patients and controls. The number of allele carriers are shown in parentheses. Rare alleles (with expected counts less than five) are combined into “others” category prior to statistical analysis. The association was examined by Chi-square test. The corrected p (P_c_) values, statistically significant after Bonferroni correction, are indicated in the bold. Abbreviations are as follows; OR, odds ratio; CI, confidence interval; P_c_, corrected p value; NS, not significant. * Separator.
(DOCX)

S3 Table Frequencies of estimated haplotype carrier in MMD patients and controls. The number of estimated haplotype carriers by the expectation maximization algorithm are shown in parentheses. Rare alleles (with expected counts less than five) are combined into “others” category prior to statistical analysis. The association was examined by Chi-square test. The corrected p (P_c_) values after Bonferroni correction are shown. Abbreviations are as follows; OR, odds ratio; CI, confidence interval; P_c_, corrected p value; NS, not significant. * Separator.
(DOCX)
