# Supplementary material for: Identification of HLA-DRB1*04:10 allele as risk allele for Japanese moyamoya disease and its association with autoimmune thyroid disease: A case-control study
Source: PLoS One. 2019 Aug 14;14(8):e0220858. doi: 10.1371/journal.pone.0220858 (PMC6693760; doi:10.1371/journal.pone.0220858)
Supplement: S2 Table — The number of allele carriers are shown in parentheses. Rare alleles (with expected counts less than five) are combined into “others” category prior to statistical analysis. The association was examined by Chi-square test. The corrected p (Pc) values, statistically significant after Bonferroni correction, are indicated in the bold. Abbreviations are as follows; OR, odds ratio; CI, confidence interval; Pc, corrected p value; NS, not significant. * Separator. (DOCX) [file pone.0220858.s002.docx]

**S2 Table Frequencies of *HLA* class** I **carrier in MMD patients and controls.**

A

| locus | Allele | Control  (2n = 814) | Patient  (2n = 272) | OR (95%CI) | P value | Pc |
| --- | --- | --- | --- | --- | --- | --- |
| A | 2:01 | 10.4 (85) | 11.0 (30) | 1.06 (0.66-1.68) | 0.785 | NS |
| A | 2:06 | 7.13 (58) | 11.0 (30) | 1.62 (0.98-2.62) | 0.041 | NS |
| A | 2:07 | 2.70 (22) | 2.21 (6) | 0.81 (0.27-2.09) | 0.654 | NS |
| A | 11:01 | 9.71 (79) | 9.19 (25) | 0.94 (0.56-1.53) | 0.803 | NS |
| A | 24:02 | 37.7 (307) | 37.9 (103) | 1.01 (0.75-1.35) | 0.964 | 0.030 |
| A | 26:01 | 7.99 (65) | 9.56 (26) | 1.22 (0.72-2.00) | 0.417 | NS |
| A | 26:03 | 2.70 (22) | 3.31 (9) | 1.23 (0.49-2.83) | 0.603 | NS |
| A | 31:01 | 7.86 (64) | 6.99 (19) | 0.88 (0.49-1.52) | 0.637 | NS |
| A | 33:03 | 8.85 (72) | 4.78 (13) | 0.52 (0.26-0.96) | 0.030 | NS |
| A | Others | 4.92 (40) | 4.05 (11) | 0.82 (0.37-1.65) | 0.557 | NS |

B

| locus | Allele | Control  (2n = 814) | Patient  (2n = 272) | OR (95%CI) | P value | Pc |
| --- | --- | --- | --- | --- | --- | --- |
| B | 7:02 | 7.00 (57) | 5.15 (14) | 0.72 (0.36-1.34) | 0.283 | NS |
| B | 15:01 | 8.60 (70) | 6.25 (17) | 0.71 (0.38-1.25) | 0.217 | NS |
| B | 35:01 | 7.99 (65) | 9.19 (25) | 1.17 (0.69-1.92) | 0.532 | NS |
| B | 39:01 | 4.18 (34) | 4.18 (7) | 0.61 (0.22-1.41) | 0.230 | NS |
| B | 40:01 | 5.53 (45) | 5.52 (15) | 1.00 (0.51-1.86) | 0.993 | NS |
| B | 40:02 | 6.76 (55) | 9.19 (25) | 1.40 (0.82-2.34) | 0.183 | NS |
| B | 40:06 | 4.05 (33) | 6.25 (17) | 1.58 (0.81-2.97) | 0.135 | NS |
| B | 44:03 | 8.11 (66) | 5.15 (14) | 0.61 (0.31-1.13) | 0.106 | NS |
| B | 46:01 | 4.67 (38) | 3.31 (9) | 0.70 (0.29-1.50) | 0.340 | NS |
| B | 48:01 | 2.58 (21) | 4.78 (13) | 1.90 (0.86-4.03) | 0.071 | NS |
| B | 51:01 | 8.23 (67) | 9.19 (25) | 1.13 (0.67-1.86) | 0.622 | NS |
| B | 52:01 | 9.71 (79) | 12.1 (33) | 1.28 (0.81-2.01) | 0.254 | NS |
| B | 54:01 | 7.62 (62) | 7.35 (20) | 0.96 (0.54-1.65) | 0.887 | NS |
| B | 55:02 | 2.46 (20) | 2.57 (7) | 1.05 (0.37-2.62) | 0.915 | NS |
| B | Others | 12.5 (102) | 9.79 (31) | 0.90 (0.57-1.39) | 0.621 | NS |

C

| locus | Allele | Control  (2n = 814) | Patient  (2n = 272) | OR (95%CI) | P value | Pc |
| --- | --- | --- | --- | --- | --- | --- |
| C | 1:02 | 16.5 (134) | 15.1 (41) | 0.90 (0.60-1.33) | 0.590 | NS |
| C | 3:03 | 13.5 (110) | 15.1 (41) | 1.14 (0.75-1.70) | 0.520 | NS |
| C | 3:04 | 12.7 (103) | 12.9 (35) | 1.02 (0.66-1.56) | 0.927 | NS |
| C | 7:02 | 14.9 (121) | 12.5 (34) | 0.82 (0.53-1.24) | 0.334 | NS |
| C | 8:01 | 5.65 (46) | 8.82 (24) | 1.62 (0.92-2.76) | 0.065 | NS |
| C | 12:02 | 9.95 (81) | 11.8 (32) | 1.21 (0.76-1.89) | 0.396 | NS |
| C | 14:02 | 5.90 (48) | 6.62 (18) | 1.13 (0.61-2.02) | 0.666 | NS |
| C | 14:03 | 8.23 (67) | 5.15 (14) | 0.60 (0.31-1.11) | 0.094 | NS |
| C | 15:02 | 3.44 (28) | 5.15 (14) | 1.52 (0.73-3.04) | 0.206 | NS |
| C | Others | 9.33 (76) | 6.99 (19) | 1.53 (0.80-2.82) | 0.149 | NS |
