# Supplementary material for: Identification of HLA-DRB1*04:10 allele as risk allele for Japanese moyamoya disease and its association with autoimmune thyroid disease: A case-control study
Source: PLoS One. 2019 Aug 14;14(8):e0220858. doi: 10.1371/journal.pone.0220858 (PMC6693760; doi:10.1371/journal.pone.0220858)
Supplement: S3 Table — The number of estimated haplotype carriers by the expectation maximization algorithm are shown in parentheses. Rare alleles (with expected counts less than five) are combined into “others” category prior to statistical analysis. The association was examined by Chi-square test. The corrected p (Pc) values after Bonferroni correction are shown. Abbreviations are as follows; OR, odds ratio; CI, confidence interval; Pc, corrected p value; NS, not significant. * Separator. (DOCX) [file pone.0220858.s003.docx]

**S3 Table Frequencies of estimated haplotype carrier in MMD patients and controls**

A

| A/B | Control  (2n = 814) | Patient  (2n = 272) | OR (95%CI) | P value | Pc |
| --- | --- | --- | --- | --- | --- |
| *02:06-*39:01 | 2.33 (19) | 1.10 (3) | 0.47 (0.09-1.60) | 0.212 | NS |
| *02:07-*46:01 | 2.33 (19) | 2.21 (6) | 0.94 (0.31-2.49) | 0.903 | NS |
| *11:01-*15:01 | 2.58 (21) | 0.74 (2) | 0.28 (0.03-1.16) | 0.067 | NS |
| *11:01-*54:01 | 2.09 (17) | 3.31 (9) | 1.60 (0.62-3.86) | 0.254 | NS |
| *24:02-*40:02 | 2.58 (21) | 2.94 (8) | 1.14 (0.43-2.73) | 0.749 | NS |
| *24:02-*51:01 | 2.33 (19) | 2.94 (8) | 1.27 (0.47-3.08) | 0.578 | NS |
| *24:02-*52:01 | 9.46 (77) | 12.1 (33) | 1.32 (0.83-2.07) | 0.206 | NS |
| *24:02-*54:01 | 3.93 (32) | 3.31 (9) | 0.84 (0.35-1.82) | 0.641 | NS |
| *26:01-*35:01 | 2.09 (17) | 1.84 (5) | 0.88 (0.25-2.51) | 0.880 | NS |
| *26:01-*40:02 | 1.97 (16) | 3.68 (10) | 1.90 (0.76-4.52) | 0.110 | NS |
| *31:01-*51:01 | 2.83 (23) | 2.94 (8) | 1.04 (0.40-2.45) | 0.921 | NS |
| *33:03-*44:03 | 7.49 (61) | 4.41 (12) | 0.57 (0.27-1.09) | 0.079 | NS |
| Others | 52.2 (425) | 53.7 (146) | 1.06 (0.80-1.41) | 0.675 | NS |

B

| A/B/C | Control  (2n = 814) | Patient  (2n = 272) | OR (95%CI) | P value | Pc |
| --- | --- | --- | --- | --- | --- |
| *02:06-*39:01-*07:02 | 2.09 (17) | 1.10 (3) | 0.52 (0.10-1.83) | 0.295 | NS |
| *02:07-*46:01-*01:02 | 2.33 (19) | 2.21 (6) | 0.94 (0.31-2.49) | 0.903 | NS |
| *11:01-*54:01-*01:02 | 1.97 (16) | 3.31 (9) | 1.71 (0.66-4.16) | 0.201 | NS |
| *24:02-*07:02-*07:02 | 5.77 (47) | 4.41 (12) | 0.75 (0.36-1.47) | 0.391 | NS |
| *24:02-*40:02-*03:04 | 1.97 (16) | 2.94 (8) | 1.51 (0.55-3.79) | 0.343 | NS |
| *24:02-*51:01-*14:02 | 1.84 (15) | 2.57 (7) | 1.41 (0.48-3.71) | 0.459 | NS |
| *24:02-*52:02-*12:02 | 9.46 (77) | 11.8 (32) | 1.28 (0.80-2.01) | 0.273 | NS |
| *24:02-*54:01-*01:02 | 3.81 (31) | 2.94 (8) | 0.77 (0.30-1.73) | 0.506 | NS |
| *26:01-*35:01-*03:03 | 2.09 (17) | 1.84 (15) | 0.88 (0.25-2.51) | 0.800 | NS |
| *26:01-*40:02-*03:04 | 1.97 (16) | 4.04 (11) | 2.10 (0.87-4.89) | 0.057 | NS |
| *31:01-*51:01-*14:02 | 1.84 (15) | 2.57 (7) | 1.41 (0.48-3.71) | 0.459 | NS |
| *33:03-*44:03-*14:03 | 7.49 (61) | 4.41 (12) | 0.57 (0.27-1.09) | 0.080 | NS |
| Others | 59.6(467) | 57.0 (152) | 0.67 (0.71-1.25) | 0.667 | NS |

C

| DRB1/DQB1/DPB1 | Control  (2n = 814) | Patient  (2n = 272) | OR (95%CI) | P value | Pc |
| --- | --- | --- | --- | --- | --- |
| *01:01-*05:01-*04:02 | 6.02 (49) | 5.15 (14) | 0.85 (0.42-1.59) | 0.594 | NS |
| *04:05-*04:01-*05:01 | 8.35 (68) | 6.25 (17) | 0.73 (0.40-1.29) | 0.263 | NS |
| *04:06-*03:02-*02:01 | 2.33 (19) | 0.74 (2) | 0.31 (0.03-1.30) | 0.097 | NS |
| *08:02-*03:02-*05:01 | 0.86 (15) | 0.74 (7) | 1.41 (0.48-3.71) | 0.459 | NS |
| *08:03-*06:01-*02:01 | 2.46 (20) | 4.78 (13) | 1.99 (0.90-4.27) | 0.053 | NS |
| *08:03-*06:01-*05:01 | 2.46 (20) | 2.21 (6) | 0.90 (0.29-2.34) | 0.815 | NS |
| *09:01-*03:03-*02:01 | 4.30 (35) | 5.88 (16) | 1.39 (0.71-2.63) | 0.286 | NS |
| *09:01-*03:03-*05:01 | 9.21 (75) | 10.7 (29) | 1.18 (0.72-1.88) | 0.482 | NS |
| *13:02-*06:04-*04:01 | 5.04 (41) | 2.94 (8) | 0.57 (0.23-1.26) | 0.149 | NS |
| *15:01-*06:02-*02:01 | 4.91 (40) | 3.31 (9) | 0.66 (0.28-1.41) | 0.270 | NS |
| *15:02-*06:01-*09:01 | 5.90 (48) | 7.72 (21) | 1.34 (0.74-2.32) | 0.286 | NS |
| Others | 53.9 (384) | 57.4 (130) | 1.03 (0.77-1.36) | 0.859 | NS |
